# Salidroside Inhibits Myogenesis by Modulating p-Smad3-Induced Myf5 Transcription

**DOI:** 10.3389/fphar.2018.00209

**Published:** 2018-03-12

**Authors:** Peng Zhang, Wenjiong Li, Lu Wang, Hongju Liu, Jing Gong, Fei Wang, Xiaoping Chen

**Affiliations:** ^1^State Key Laboratory of Space Medicine Fundamentals and Application, China Astronaut Research and Training Center, Beijing, China; ^2^National Key Laboratory of Human Factors Engineering, China Astronaut Research and Training Center, Beijing, China

**Keywords:** salidroside, myogenesis, Myf5, p-Smad3, myoblast, reserve cell

## Abstract

**Aim:** Salidroside is an active compound extracted from *Rhodiola rosea* which is used to alleviate fatigue and enhance endurance in high altitude regions. Some studies have demonstrated that salidroside can affect precursor cell differentiation in hematopoietic stem cells, erythrocytes, and osteoblasts. The aim of this study was to investigate the effect of salidroside on myoblast differentiation and to explore the underlying molecular mechanisms of this effect.

**Methods:** C2C12 myoblast cells were treated with different concentrations of salidroside in differentiation media. Real-time PCR, Western blotting, and immunofluorescence assay were employed to evaluate the effects of salidroside on C2C12 differentiation. RNA interference was used to reveal the important role of Myf5 in myogenesis inhibited by salidroside. Chromatin Immunoprecipitation and dual-luciferase reporter assay were utilized to explore the underlying mechanisms of salidroside-induced upregulation of Myf5.

**Results:** We found that salidroside inhibits myogenesis by downregulating MyoD and myogenin, preserves undifferentiated reserve cell pools by upregulating Myf5. Knocking down Myf5 expression significantly rescued the myogenesis inhibited by salidroside. The effect of salidroside on myogenesis was associated with increased phosphorylated Smad3 (p-Smad3). Both SIS3 (Specific inhibitor of p-Smad3) and dominant negative Smad3 plasmid (DN-Smad3) attenuated the inhibitory effect of salidroside on C2C12 differentiation. Moreover, the induction of Myf5 transcription by salidroside was dependent on a Smad-binding site in the promoter region of Myf5 gene.

**Conclusion and Implications:** Our findings identify a novel role and mechanism for salidroside in regulating myogenesis through p-Smad3-induced Myf5 transcription, which may have implications for its further application in combating degenerative muscular diseases caused by depletion of muscle stem cells, such as Duchenne muscular dystrophy or sarcopenia.

## Introduction

Myogenesis, the formation of muscular tissue, occurs during embryonic development, postnatal growth, and adult muscle regeneration (Tajbakhsh, 2009; Ryall, 2013). In vertebrates, myogenesis is controlled by the MRFs, including Myf5, MyoD, myogenin, and MRF4 (Dhawan and Rando, 2005; Yokoyama and Asahara, 2011; Moncaut et al., 2013). Upon muscle injury, satellite cells, the main stem cells located between the muscle fiber and the basal lamina, withdraw from their quiescent state and differentiate into myoblasts with upregulated expression of Myf5. Most of the myoblasts continue to differentiate with high expression of MyoD and myogenin, and fuse into the injured area to repair the damaged fibers. However, a fraction of the satellite cells do not differentiate but self-renew to form a reservoir of satellite cells. Similarly, undifferentiated C2C12 myoblasts have also been observed after terminal differentiation *in vitro* and were named as reserve cells (Yoshida et al., 1998). Recent studies have demonstrated that Myf5 is essential for the maintenance of the satellite cell pool, which plays a pivotal role in the regenerative capacity of adult muscle tissue (Gunther et al., 2013).

To date, several pathways that regulate myogenesis have been identified, and among these, the TGF-β/Smad pathway is a key negative regulator of myogenesis (Kollias and McDermott, 2008; MacDonald and Cohn, 2012). TGF-β1 and its family members, such as myostatin, inhibit the differentiation, fusion, and myotube formation of primary myoblasts and C2C12 myoblasts. The downstream transcription factor, Smad3, mediates most of the effects of TGF-β on myogenesis. p-Smad3 targets and represses the expression of MyoD and myogenin by binding to their bHLH domain (Liu et al., 2001; Langley et al., 2002; McFarlane et al., 2011). Smad3 is also capable of repressing myogenin expression by interacting with MEF2. Therefore, the TGF-β/Smad3 pathway may provide an attractive target for therapeutic intervention of degenerative muscular diseases.

Salidroside is a phenylpropanoid glycoside extracted from the medicinal plant *Rhodiola rosea* and commonly used in traditional Tibetan medicine to battle fatigue and enhance exercise performance (Darbinyan et al., 2000; Spasov et al., 2000; Shevtsov et al., 2003). Recently, several studies have demonstrated that salidroside or extracts from *Rhodiola rosea* can affect precursor cell differentiation in several cell types. In HSCs, salidroside protects erythrocytes from hydrogen peroxide-induced apoptosis and promotes erythropoiesis (Qian et al., 2011, 2012). Li et al. (2012a) demonstrated that salidroside prevents the loss of HSCs under oxidative stress by activating poly (ADP-ribose) polymerase-1 (PARP-1) to reduce DNA-strand breaks and that the process of recruiting quiescent HSCs into the cell cycle was blocked (Li et al., 2012a). *Rhodiola crenulata* extract, of which salidroside is the main ingredient, improves impaired neurogenesis in the hippocampus of depressed rats and in rats with streptozotocin-induced neural injury (Chen et al., 2009; Qu et al., 2012). Salidroside also has been proven to stimulate osteoblast differentiation through BMP signaling pathway (Chen et al., 2013). However, there has been no report regarding the effect of salidroside on the myogenic differentiation process.

In the present study, we investigated the effect of salidroside on myogenesis using C2C12 myoblasts *in vitro*. For the first time, we report that salidroside efficiently inhibits the myogenic differentiation of C2C12 cells *in vitro*, mainly by enhancing p-Smad3-induced Myf5 transcription. Our results also delineate a direct regulatory mechanism between Smad3 signaling and Myf5 transcription.

## Materials and Methods

### Cell Culture

C2C12 myoblast cells were cultured as described previously (Li et al., 2012b). To induce myogenesis *in vitro*, proliferating myoblasts were shifted from GM (high-glucose DMEM supplemented with 10% FBS, 100 units/ml penicillin, 0.1 mg/ml streptomycin, 20 mM glutamine) into DM (high-glucose DMEM supplemented with 2% horse serum, 100 units/ml penicillin, 0.1 mg/ml streptomycin, 20 mM glutamine). To observe the influence of salidroside (Chengdu Herbpurify, Co., Ltd., purity ≥ 99%, H-040-110826) on myogenesis, C2C12 cells were treated with three concentrations of salidroside (25, 50, 100 μg/ml) in DM for 120 h. For the time-courses assay, C2C12 cells were harvested at 24, 48, 72, 96, or 120 hours (h) after treatment of 50 μg/ml of salidroside. In some cases, C2C12 cells were pretreated in serum-free medium with 5 μM SIS3 (Calbiochem, 566405) for 60 min in advance and then incubated with 50 μg/ml of salidroside for 120 h. Control cells received equivalent amounts of vehicle (DMSO).

### Cell Viability

Cell viability was assessed by MTT assay. C2C12 cells were treated with different concentrations of salidroside (25, 50, 100 μg/ml) in DM and control cells were added with equal volume of PBS. After 120 h incubation, methyl-thiazolyl-tetrazolium (MTT) was added to a final concentration of 0.5 mg/ml and cells were incubated for another 3 h in a humidified 5% CO_2_ incubator at 37°C. Next, medium was aspirated, 100 μl DMSO was added, and absorbance was read out at 580 nm.

### Vector Construction and Transfection

For promoter assay, a Myf5 reporter plasmid was constructed by ligating 1000 bp of the murine genomic region upstream of the 5^′^ UTR of the Myf5 gene into the pGL3-Basic reporter (Promega, E1751) to generate the pGL3-Myf5-wt plasmid. The Myf5 genomic fragment was generated by PCR using the following primers, forward 5^′^-ACCGAGCTC TTACGCGTGCTAGCTGTCACCAAAGTGTGTGAAGCCACTCT-3^′^, and reverse 5^′^-TTAGATCGCAGATCTCGAGGGAGGTTGGTCCCTGTAGCTGGG-3^′^. To mutate the potential Smad-binding site in the promoter region, overlapping PCR was performed with two additional primers (5^′^-GAGGCTTGCCCTTTTTTCCCCTGTGGGGGGTTGTGGTGGGAT-3^′^ and 5^′^-ACCCCCCACAGGGGAAAAAAGGGCAAGCCTCTTGTCTTTCTTCTAGAGAC-3^′^) to generate the pGL3-Myf5-mut plasmid. A dominant negative Smad3 plasmid (DN-Smad3) was constructed as described previously (Park et al., 2000). Plasmids were transfected into C2C12 cells using the NeonTM Transfection System according to the manufacturer’s instructions (Invitrogen, MPK5000).

### Luciferase Assay

Smad-responsive CAGA luciferase reporter was purchased from Promega (E367A). For luciferase assays, cells were co-transfected with 500 ng of reporter plasmids, 25 ng of pRL-TK vectors (Promega, E2231) as an internal control. Luciferase activity was analyzed using the dual-luciferase reporter assay system (Promega, E1910) and measured with a Glomax^™^ Detection System (Promega, E6080) by following the manufacturer’s instructions. Three independent experiments were carried out in duplicate.

### RNA Interference

C2C12 myoblasts were plated at 50–60% confluence in six well culture plates and incubated for 24 h. For each transfection, cells were transfected with siRNA (Santa Cruz, sc-35989) targeting Myf5 or control siRNA (Santa Cruz, sc-36869) using X-tremeGene siRNA transfection reagent (Roche, 4476093001) according to the manufacturer’s protocol. After 6 h, the transfection medium was replaced by normal GM for 24 h. Then, the C2C12 cells were induced to differentiate as described above.

### Immunoblotting and Immunofluorescence Assay

C2C12 cells were lysed in RIPA buffer (50 mM Tris-HCl, 150 mM NaCl, 1 mM EDTA, 1% Triton X-100, and 1% protease) with phosphatase inhibitors (Roche, 04906845001). Supernatants were collected and the protein concentration was determined using the Bradford protein assay reagent (Bio-Rad, 500-0203). Equal amounts of extracted proteins (30 μg per lane) were denatured in sodium dodecyl sulfate (SDS) loading buffer, centrifuged briefly to remove insoluble material, and separated on SDS-PAGE. The protein was then transferred onto a nitrocellulose membrane, which was blocked in 5% non-fat milk or bovine serum albumin diluted in Tris-buffered saline-Tween for 1 h and then incubated overnight at 4°C with the following primary antibodies against Smad3 (Abcam, ab40854), p-Smad3 (Abcam, ab52903), MyoD (Abcam, ab64159), Myf5 (Abcam, ab125301), myogenin (Abcam, ab124800), or beta-actin (Santa Cruz, sc130656). For IFA, C2C12 cells were fixed with 4% formaldehyde for 30 min at 4°C and then treated with 0.5% Triton X-100 in PBS for 5 min at room temperature. After that, the cells were incubated with a primary antibody against E-MHC (Hybridoma Bank, BF-G6), p-Smad3, Myf5 or myogenin at 4°C overnight (1:200 dilutions), followed by incubation with the Alexa Fluor 594 (Invitrogen, A-11032) fluorescent dye conjugated to an anti-mouse secondary antibody or Alexa Fluor 488 (Invitrogen, A-11034) fluorescent dye conjugated to an anti-rabbit secondary antibody. The cells were stained with DAPI to visualize the nuclei. Photo capture was performed using a Nikon laser microscope (Eclipse E600, Nikon Instruments, Inc., Japan). For each sample, more than eight fields per dish were picked. The fluorescence areas, the number of fluorescence-positive nuclei and total nuclei with DAPI staining were counted with Image-Pro plus 6.0 (Media Cybernetics, Inc., United States).

### RNA Extraction and Real-Time PCR

Total RNA was extracted with TRIzol reagent according to the manufacturer’s protocol (Invitrogen, 15596-026), and a preamplification system was used to reverse transcribe the total RNA (2 μg) into complementary DNA according to the manufacturer’s instructions (Takara, RR037A). Real-time PCR was performed using a StepOnePlus Realtime PCR system (Invitrogen, 4376592) with Fast SYBR^™^ Green Master Mix (ABI, 4385612). The following primers were used: Myf5, forward 5^′^-CTCAGGAATGCCATCCGCTA-3^′^ and reverse 5^′^-CGGATGGCTCTGTAGACGTG-3^′^; MyoD, forward 5^′^-CGGCTCTCTCTGCTCCTTTG-3^′^ and reverse 5^′^-GAGTCGAAACACGGGTCATCA-3^′^; myogenin, forward 5^′^-GACCCTACAGACGCCCACAA-3^′^ and reverse 5^′^-CCGTGATGCTGTCCACGAT-3^′^; and GAPDH, forward 5^′^-GGAAGCTTGTCATCAACGGG-3^′^ and reverse 5^′^-GGCGGAGATGATGACCCTTTT-3^′^. Optimal PCR conditions were determined for all primers. Each PCR mixture (final reaction volume, 50 μl) contained 21 μl of sterile water, 25 μl SYBR Green reaction mix, 2 μl of cDNA (500 ng/μl), 1 μl of forward primer (10 pmol/μl), 1 μl of reverse primer (10 pmol/μl). PCR was performed with an initial denaturation protocol at 95°C for 10 min, followed by 40 cycles of denaturation at 95°C for 10 s, annealing according to the melting temperature of the specific primer for 15 s, elongation at 72°C for 20 s, and finally concluding with a melting curve step. The expression levels of target genes were normalized to GAPDH.

### ChIP Assay

Chromatin Immunoprecipitation assays were performed by using a Chromatin Immunoprecipitation Kit from Millipore (17-295) according to the manufacturer’s instructions. Briefly, after 120 h of differentiation, C2C12 treated with or without salidroside were crosslinked with 1% formaldehyde. Cells were then lysed and the chromatin was harvested and fragmented using sonication. The samples were precleared with Protein G agarose beads and immunoprecipitated using a ChIP-grade antibody to Smad3 (Abcam, ab28379), or an equal amount of control IgG (Cali-Bio, CB200071) in the presence of salmon sperm DNA. Beads were washed extensively before reverse crosslinking. DNA was purified to remove the chromatin proteins. The genomic region of Myf5 flanking the potential Smad-binding site was amplified with the following primer pair; forward 5^′^-AGGTTAGGCTGCAGTAAAATCA-3^′^ and reverse 5^′^-GGAGGTTGGTCCCTGTAGCTGG-3^′^.

### Statistical Analysis

Data are presented as the means ± SEM. The two-tailed Student’s *t*-test was used for comparison between two groups, and multi-group comparisons were performed with the one-way ANOVA test followed by Bonferroni *post hoc* test using GraphPad Prism version 5.0 (GraphPad Software, Inc.). *P*-values of less than or equal to 0.05 were considered statistically significant.

## Results

### Salidroside Efficiently Inhibits the Differentiation of C2C12 Myoblasts

**FIGURE 1:**
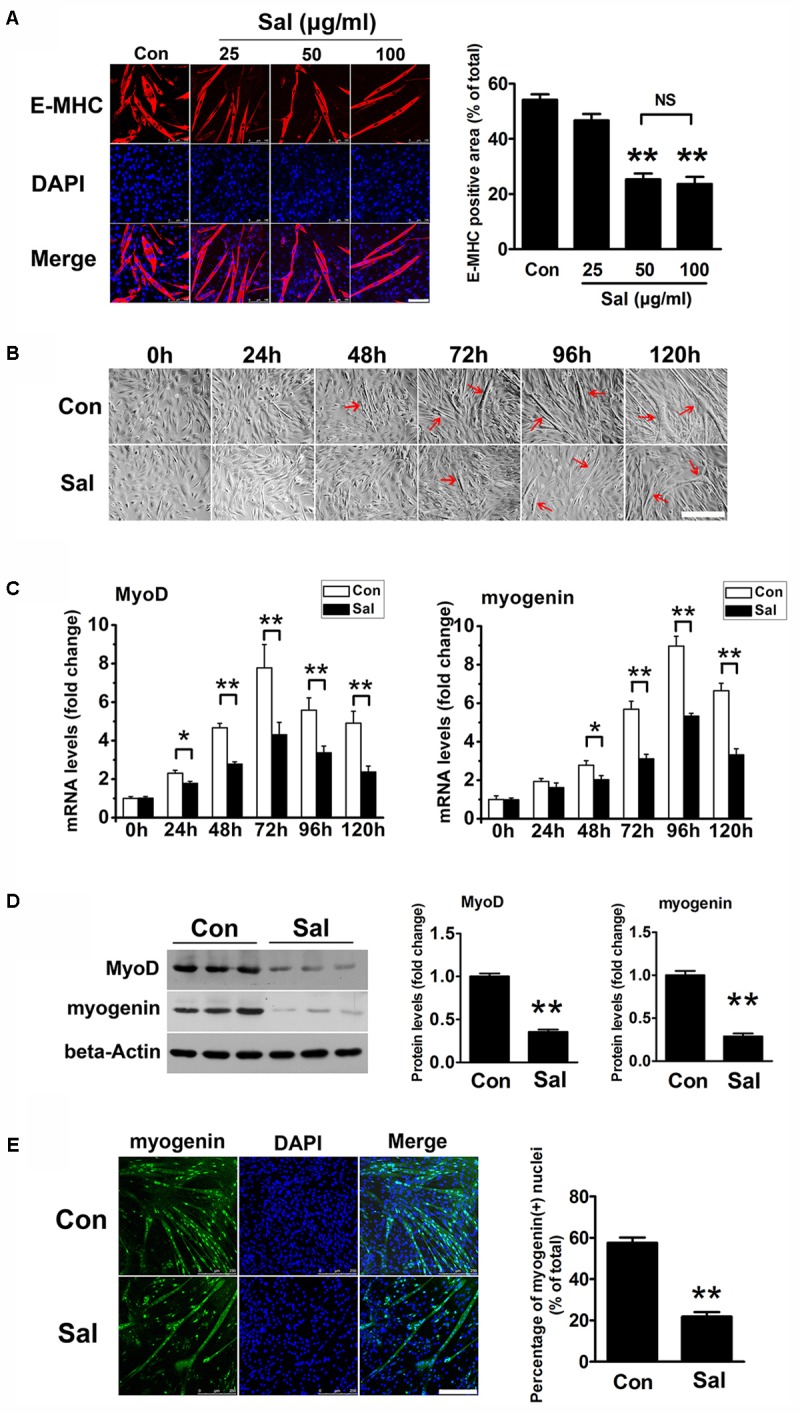
Salidroside inhibits myoblast differentiation. **(A)** C2C12 cells treated with or without different concentrations of salidroside (Sal; 25, 50, 100 μg/ml) in differentiation media (DM) for 120 h were fixed and immunostained with anti-embryonic myosin heavy chain antibody. Images were taken with the same camera setting and exposure time. Scale bar: 100 μm. The E-MHC positive area was quantified by Image-Pro Plus 6.0 software (*n* = 6). NS, not significant. **(B)** Light microscopy showing myotube formation in cell samples cultured in DM with (Sal) or without (Con) 50 μg/ml salidroside for 0∼120 h. Scale bar: 250 μm. Myotubes were indicated with red arrows. **(C)** Real-time PCR analysis of MyoD and myogenin mRNA expression in C2C12 cells treated with or without 50 μg/ml salidroside in DM for 0∼120 h (*n* = 6). **(D)** Western blotting analysis of MyoD and myogenin expression levels in C2C12 cells treated with or without 50 μg/ml salidroside in DM for 120 h (*n* = 6). The intensity of the protein bands was quantified by densitometry with Image-Pro Plus 6.0 software. **(E)** C2C12 cells treated with or without 50 μg/ml salidroside in DM for 120 h were fixed and immunostained with an anti-myogenin antibody. Scale bar: 250 μm. The myogenin positive nuclei were quantified by Image-Pro Plus 6.0 software (*n* = 6). All data are shown as the means ± SEM. ^∗^*p* < 0.05, ^∗∗^*p* < 0.01 compared with control group by one-way ANOVA **(A)** or Student’s *t*-test **(C–E)**.

To investigate the effect of salidroside on myogenesis *in vitro*, we treated C2C12 cells with varying concentrations of salidroside (25, 50, or 100 μg/ml) in DM and analyzed their ability to undergo myogenic differentiation. Cytotoxicity of each concentration of salidroside was assessed in parallel using the MTT assay. As shown in **Supplementary Figure S1**, salidroside had no cytotoxicity on C2C12 cells during differentiation. After 120 h of differentiation, control C2C12 cells showed extensive formation of multinucleated myotubes, which were characterized by the expression of E-MHC, a structural protein specific to myotubes (**Figure 1A**). By contrast, C2C12 cells treated with 50 or 100 μg/ml salidroside formed less myotubes than the control cells, with low levels of E-MHC (**Figure 1A**). A lower concentration (25 μg/ml) of salidroside had a slight but non-significant effect on myotube formation and E-MHC expression (**Figure 1A**). By comparing the morphology of myotubes treated with or without 50 μg/ml salidroside at different time points, we observed a delayed myotube formation in the salidroside-treated cells. Control C2C12 cells began to form typical myotubes approximately 48–72 h after differentiation, but myotube formation was delayed in the salidroside-treated cells beginning 72–96 h after differentiation (**Figure 1B**). Next, we examined the effect of salidroside on the expression of MRFs, MyoD and myogenin. MyoD and myogenin expression levels were dramatically upregulated in control cells during differentiation. In contrast, this upregulation was dramatically reduced in cells treated with salidroside (**Figure 1C**). In agreement with the changes in gene expression, MyoD and myogenin protein expression levels were also inhibited in myotubes treated with salidroside (**Figure 1D**), and the number of myogenin-positive nuclei was also reduced (**Figure 1E**). These findings indicate that salidroside inhibits myogenic differentiation in a dose- and time-dependent manner.

### Salidroside Increases the Myf5-Positive Cells Pool *in Vitro*

**FIGURE 2:**
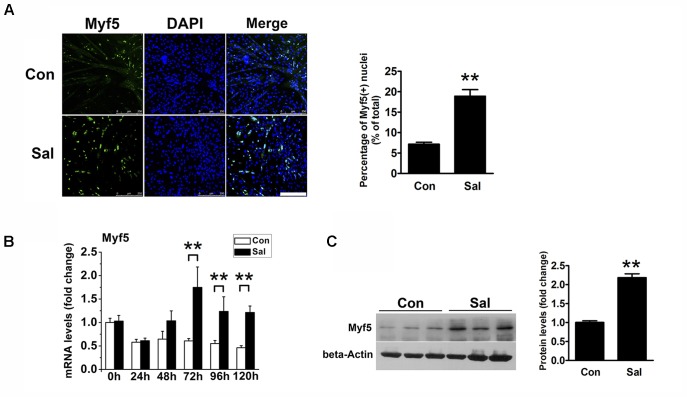
Salidroside preserves the reserve cell pool *in vitro*. **(A)** C2C12 cells treated with or without 50 μg/ml salidroside in DM for 120 h were fixed and immunostained with anti-Myf5 antibody. Scale bar: 250 μm. The Myf5 positive nuclei were quantified by Image-Pro Plus 6.0 software (*n* = 6). **(B)** Real-time PCR analysis of Myf5 mRNA expression in C2C12 cells treated with or without 50 μg/ml salidroside in DM for 0∼120 h (*n* = 6). **(C)** Western blotting analysis of Myf5 expression in C2C12 cells treated with or without 50 μg/ml salidroside in DM for 120 h (*n* = 6). The intensity of the protein bands was quantified by densitometry with Image-Pro Plus 6.0 software. All data are shown as the means ± SEM. ^∗∗^*p* < 0.01 compared with the control group by Student’s *t*-test.

We noticed that there were more undifferentiated cells surrounding the salidroside-treated myotubes than the control myotubes after 120 h of differentiation, which raised the possibility that salidroside could increase the pool of reserve cells. To test this possibility, we detected the Myf5-positive cells surrounding myotubes using IFA. As shown in **Figure 2A**, there were only a few Myf5-positive cells around the control myotubes, indicating that most of the myoblasts had differentiated into myotubes. In contrast, more Myf5-positive cells were observed surrounding the salidroside-treated myotubes. Consistent with the IFA results, Myf5 mRNA was downregulated in the control cells throughout the course of differentiation; however, the expression of Myf5 mRNA was increased in the salidroside-treated cells during differentiation (**Figure 2B**). The Myf5 protein expression was also upregulated in myotubes treated with salidroside for 120 h (**Figure 2C**). These data demonstrate that salidroside preserves the Myf5-positive reserve cells after terminal differentiation.

### Myf5 Plays a Pivotal Role in the Effect of Salidroside on C2C12 Differentiation

**FIGURE 3:**
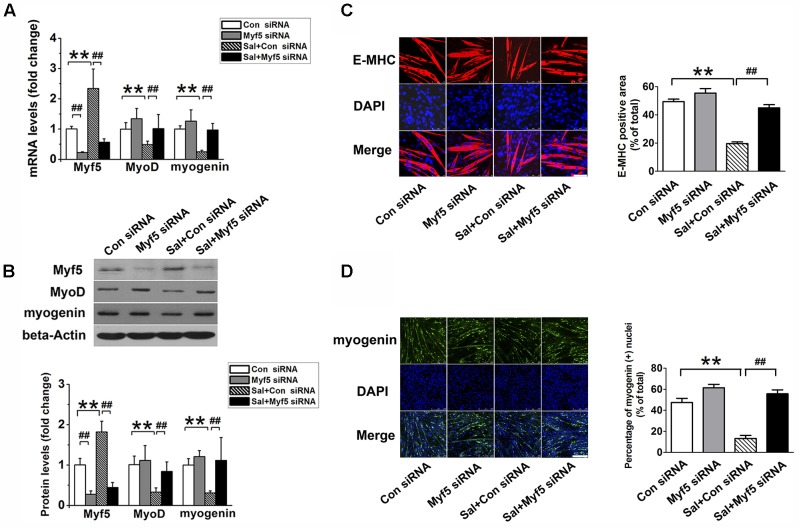
Myf5 knockdown rescues the impaired differentiation of salidroside-treated cells. **(A)** C2C12 cells were either transfected with control siRNA or Myf5 siRNA and then treated with 50 μg/ml salidroside in DM for 120 h. Total RNA was then extracted and the levels of Myf5, MyoD, and myogenin mRNA were quantitated by Real-time PCR analysis (*n* = 6). **(B)** Western blotting analysis of Myf5, MyoD, and myogenin expression levels in C2C12 cells treated as described in **(A)**. The intensity of the protein bands was quantified by densitometry with Image-Pro Plus 6.0 software (*n* = 6). **(C)** C2C12 cells treated as described in **(A)** were subjected to immunofluorescence staining by using an anti-E-MHC antibody. Images were taken with the same camera settings and exposure time. Scale bar: 75 μm. The E-MHC positive area was quantified by Image-Pro Plus 6.0 software (*n* = 6). **(D)** C2C12 cells treated as described in **(A)** were subjected to immunofluorescence staining by using an anti-myogenin antibody. Scale bar: 250 μm. The myogenin positive nuclei were quantified by Image-Pro Plus 6.0 software (*n* = 6). All data are shown as means ± SEM. ^∗∗^*p* < 0.01, ^##^*p* < 0.01 compared with groups as indicated by one-way ANOVA.

To determine whether the upregulation of Myf5 was responsible for the delayed myogenic differentiation induced by salidroside, we transfected C2C12 cells with control or Myf5-specific siRNA and, then incubated transfected cells with or without salidroside in DM for 120 h. Real-time PCR and Western blotting results confirmed the knockdown of Myf5 expression in C2C12 myotubes transfected with Myf5 siRNA (**Figures 3A,B**). Although salidroside impaired myogenic differentiation in cells transfected with control siRNA, as demonstrated above, more myotubes were easily observed in the salidroside-treated cells transfected with Myf5 siRNA (**Figure 3C**). The expression of E-MHC recovered to those of the control cells (**Figure 3C**). Both the gene and protein expression levels of MyoD and myogenin in salidroside-treated cells transfected with Myf5 siRNA were significantly upregulated and reached levels comparable to those of the control cells (**Figures 3A,B,D**). The number of myogenin-positive nuclei also recovered to those of the control cells (**Figure 3D**). Collectively, these data demonstrate that the interference of Myf5 expression could rescue the delayed differentiation of salidroside-treated cells.

### Salidroside Activates Phospho-Smad3 in C2C12 Myotubes

**FIGURE 4:**
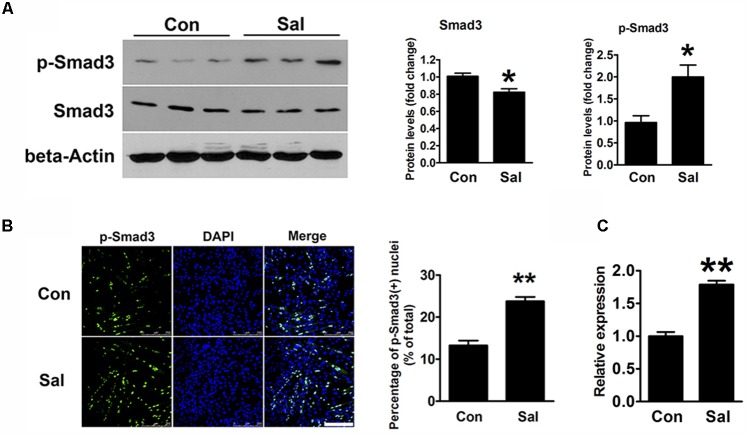
Salidroside activates the TGF-β1/Smad3 signaling pathway during myogenic differentiation. **(A)** Western blotting analysis of Smad3 and p-Smad3 expression levels in C2C12 cells treated with or without 50 μg/ml salidroside in DM for 120 h. The intensity of the protein bands was quantified by densitometry with Image-Pro Plus 6.0 software (*n* = 6). **(B)** C2C12 cells treated as described in **(A)** were subjected to immunofluorescence using an anti-p-Smad3 antibody (*n* = 6). Scale bar: 250 μm. The p-Smad3 positive nuclei were quantified by Image-Pro Plus 6.0 software. **(C)** C2C12 cells transfected with Smad-responsive luciferase reporter were treated with or without 50 μg/ml salidroside in DM. Luciferase assays were performed with the cell extracts; a Renilla luciferase vector cotransfected was used to normalize the transfection efficiency (*n* = 6). All data are shown as the means ± SEM. ^∗^*p* < 0.05, ^∗∗^*p* < 0.01 compared with the control group by Student’s *t*-test.

TGF-β/Smad3 signaling is a well-known negative regulatory pathway of myogenesis. To explore the signaling mechanisms involved in the effect of salidroside on myogenesis, we detected the protein expression of Smad3 and p-Smad3 by using Western blotting. Compared to the levels in the control cells, p-Smad3 was significantly increased, whereas unphosphorylated Smad3 was decreased by salidroside treatment (**Figure 4A**). IFA also showed that the amount of p-Smad3 located in the nuclei was significantly increased in the salidroside treated group (**Figure 4B**), suggesting the activation of the TGF-β/Smad3 signaling pathway by salidroside in muscle cells. In addition, we transfected C2C12 cells with Smad-responsive reporter plasmid, and then incubated transfected cells with salidroside in DM for 120 h. As shown in **Figure 4C**, the luciferase activity of Smad-responsive reporter plasmid was induced by salidroside. These data suggest that salidroside is capable of activating TGF-β/Smad3 signaling pathway in muscle cells.

### Specific Blocking of Smad3 Signaling Rescued the Inhibitory Effect of Salidroside on Myogenesis

**FIGURE 5:**
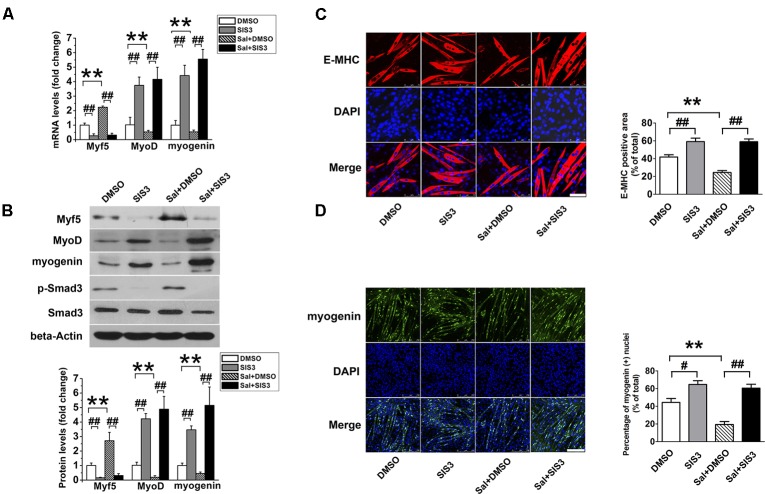
SIS3 rescues the inhibitory effect of salidroside on C2C12 differentiation. **(A)** Real time-PCR analysis of Myf5, MyoD, and myogenin expression levels in C2C12 cells pretreated with vehicle (DMSO) or with 5 μM SIS3 for 60 min prior to an incubation with 50 μg/ml salidroside in DM for 120 h. Total RNA was extracted from the cells, and the expression levels of Myf5, MyoD, and myogenin were quantitated by real-time PCR analysis (*n* = 6). **(B)** Western blotting analysis of Smad3, p-Smad3, Myf5, MyoD, and myogenin expression in C2C12 cells treated as described in **(A)**. The intensity of the protein bands was quantified by densitometry with Image-Pro Plus 6.0 software (*n* = 6). **(C)** C2C12 cells treated as described in **(A)** were subjected to immunofluorescence staining by using an anti-E-MHC antibody. Images were taken with the same camera settings and exposure time. Scale bar: 75 μm. The E-MHC positive signal was quantified by Image-Pro Plus 6.0 software (*n* = 6). **(D)** C2C12 cells treated as described in **(A)** were subjected to immunofluorescence staining by using an anti-myogenin antibody. Scale bar: 250 μm. The myogenin positive nuclei were quantified by Image-Pro Plus 6.0 software (*n* = 6). All data are shown as the means ± SEM. ^∗∗^*p* < 0.01, ^#^*p* < 0.05, ^##^*p* < 0.01 compared with groups as indicated by one-way ANOVA.

SIS3 is a Smad3-specific inhibitor. To verify whether Smad3 signaling mediates the inhibitory effect of salidroside on myogenic differentiation, C2C12 cells were incubated with 5 μM SIS3 or DMSO for 60 min prior to exposure to 50 μg/ml salidroside in DM; then, the expression levels of MRFs and the activation state of the Smad3 signaling pathway were examined. Western blotting confirmed that SIS3 abrogated Smad3 phosphorylation (**Figure 5B**). SIS3 pretreatment enhanced myotube formation and increased the expression levels of MyoD and myogenin both in the salidroside-treated and non-treated groups (**Figures 5A,B**), supporting the well-established inhibitory role of Smad3 on myogenic differentiation. Salidroside impaired myogenic differentiation in cells pretreated with DMSO, but more myotubes were easily observed in salidroside-treated cells pretreated with SIS3 and the expression of E-MHC was recovered to those of the control cells (**Figure 5C**). Both the gene and protein expression levels of MyoD and myogenin in the salidroside-treated cells were upregulated significantly when Smad3 was inhibited and reached levels comparable to those of the control cells (**Figures 5A,B,D**). Interestingly, the expression of Myf5 was sharply downregulated by the inhibition of Smad3 signaling in both the control and salidroside-treated groups (**Figures 5A,B**), suggesting that Smad3 may mediate the salidroside-induced upregulation of Myf5. To further confirm the involvement of Smad3 in mediating the effect of salidroside on myogenesis, cells were transfected with a dominant negative Smad3 plasmid (DN-Smad3) before incubating with or without salidroside in DM for 120 h. As shown in **Supplementary Figure S2**, expression of DN-Smad3 completely reversed the effect of salidroside on myogenesis.

### A Smad Binding Site in the Myf5 Promoter Is Essential for the Enhancement of Myf5 Expression by Salidroside

**FIGURE 6:**
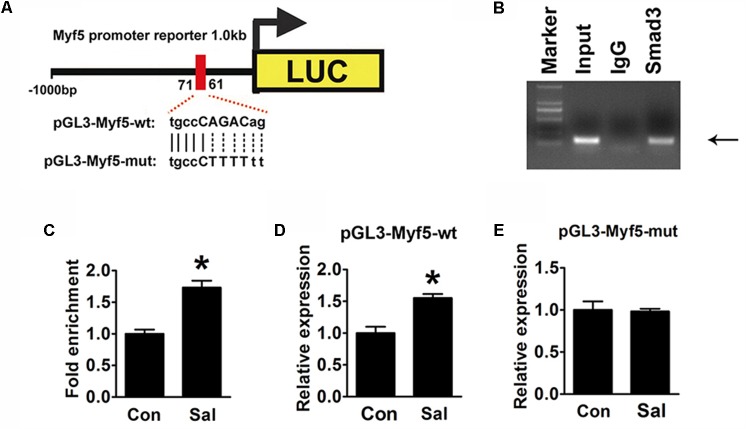
Induction of Myf5 transcription expression by salidroside depends on a Smad-binding site. **(A)** A schematic illustration of the native (pGL3-Myf5-wt) and mutated sequences (pGL3-Myf5-mut) of the potential Smad-binding site in the Myf5 promoter. **(B)** The ChIP assay was performed using C2C12 cells and revealed Smad3 binding on the Myf5 promoter. Sonicated chromatin extracted from C2C12 cells was immunoprecipitated with antibodies against IgG, or Smad3. Primers flanking the potential Smad-binding site on the Myf5 promoter were used for amplifying DNA by PCR (*n* = 6). **(C)** The ChIP assay was performed on C2C12 myotubes treated with or without salidroside in DM. Sonicated chromatin extracted from myotubes was immunoprecipitated with Smad3 and then analyzed by real-time PCR using primers as in **(B)**. Values indicate the relative enrichment of Smad3 at the putative Smad-binding site on the proximal Myf5 promoter (*n* = 6). **(D,E)** C2C12 cells transfected with either the pGL3-Myf5-wt or pGL3-Myf5-mut plasmid were treated with or without 50 μg/ml salidroside in DM. Luciferase assays were performed with the cell extracts; a Renilla luciferase vector cotransfected was used to normalize the transfection efficiency (*n* = 6). All data are shown as the means ± SEM. ^∗^*p* < 0.05 compared with control group by Student’s *t*-test.

*In silico* analysis^1^ suggested that a putative conserved Smad binding site (tgccCAGACag) existed between -71∼-61 bp in the Myf5 promoter region (**Figure 6A**). This indicated that the site might be the element by which p-Smad3 directly regulates Myf5. To prove this, a ChIP assay was performed to examine the interaction of p-Smad3 with the Myf5 promoter region flanking this site. A ChIP-grade antibody against Smad3 successfully immunoprecipitated the potential Smad3-binding site of the Myf5 promoter region (**Figure 6B**), supporting a physical interaction between Smad3 and the Myf5 promoter region. Moreover, ChIP assays with sonicated chromatin extracted from C2C12 myotubes confirmed the increased binding of Smad3 to the promoter region of the Myf5 gene in salidroside treated myotubes (**Figure 6C**).

To determine if this binding site was indispensable for the induction of Myf5 transcription by salidroside, we constructed luciferase reporter plasmids containing either the 1000 bp genomic DNA fragment upstream of the Myf5 gene (pGL3-Myf5-wt) to drive the expression of a luciferase reporter gene or a mutant version of this promoter (pGL3-Myf5-mut) generated by mutating the Smad binding site (**Figure 6A**). The luciferase activity of the pGL3-Myf5-wt promoter was increased in the cells treated with salidroside (**Figure 6D**). However, when C2C12 cells were transfected with the pGL3-Myf5-mut plasmid, salidroside failed to increase the transcription of Myf5 (**Figure 6E**). These results demonstrate that salidroside induce Myf5 expression relying on the binding of p-Smad3 in the conserved binding site of the Myf5 promoter region.

## Discussion

Salidroside is the main ingredient isolated from the medicinal plant *Rhodiola rosea* which has been widely used as a folk medicine in Asian–European countries including China, Uzbekistan, France, and Germany for centuries. Due to its effective anti-fatigue properties and its enhancement of physical and mental performance, salidroside is one of the few phytotherapies that prevents altitude sickness and increases endurance at high altitudes or cold regions. Some researchers even regard salidroside as an adaptogen based on its broad spectrum of pharmacological properties (Guo et al., 2010; Mao et al., 2010; Bayliak and Lushchak, 2011; Li et al., 2011; Chen et al., 2012). Surprisingly, except for its anti-fatigue and endurance-enhancing properties (Abidov et al., 2003; Li H.B. et al., 2008; Zhang et al., 2013), few effects of salidroside or *Rhodiola rosea* on skeletal muscle have been recognized. The results of this study demonstrate a new function of salidroside in regulating myogenic differentiation *in vitro*. Specifically, we have shown that (1) salidroside is sufficient to inhibit myogenic differentiation by preserving the Myf5-positive cell pool, (2) p-Smad3 is a mediator of salidroside in muscle cells, and (3) p-Smad3 regulates Myf5 directly and contributes to the effect of salidroside on myogenesis. To the best of our knowledge, this is the first report on the effect of salidroside on myogenesis, a process that not only occurs in early development, but also serves as a continuous remodeling program in adults during regeneration.

When damage is induced by eccentric exercise or pathological conditions such as in the muscular dystrophies, skeletal muscle undergoes a vigorous regenerative response. Satellite cells located between the muscle fiber and the basal lamina are regarded as the main stem cells responsible for adult muscle repair. Although the pool of satellite cells is heterogeneous in terms of origin (Biressi and Rando, 2010), most satellite cells resident in adult hindlimb muscles derive from cells expressing Myf5 at a fetal stage (Biressi et al., 2013). Approximately, 90% of quiescent satellite cells in the adult muscles are positive for Myf5 (Gayraud-Morel et al., 2012). Upon muscle injury, satellite cells upregulate the expression of MyoD and myogenin and fuse into damaged myofibers, contributing cytoplasm and new nuclei to the myofiber structure and accounting for almost half of the force restored after contraction-induced injuries (Ambrosio et al., 2009). Meanwhile, a small portion of satellite cells self-renew to create a new residual pool of satellite cells for future use (Gunther et al., 2013). Normally, a relatively small number of Myf5 positive satellite cells are sufficient for efficient repair of skeletal muscles (Gunther et al., 2013). Myf5-deficient mice display reduced muscle mass and a delay of skeletal muscle regeneration (Ustanina et al., 2007). Loss of Myf5 in *mdx* mice accelerates the dystrophic changes and impairs the continuous regeneration of myofibers that occurs in *mdx* mice (Ustanina et al., 2007). In contrast, the elevated expression of Myf5 compensates for the loss of MyoD and maintains a normal muscle development in MyoD-deficient mice (Rudnicki et al., 1992). Therefore, increasing the Myf5 positive satellite cell pool is a plausible strategy to enhance muscle regenerative capacity and combat some degenerative diseases caused by physiological aging or muscular dystrophies. For example, catechin has been reported to activate satellite cells by induction of Myf5 transcription and stimulate muscle regeneration (Kim et al., 2017). In our study, for the first time, we detected the effect of salidroside on Myf5 expression and the number of Myf5 positive cells after terminal differentiation. We found that salidroside is capable of increasing the Myf5-positive cells *in vitro* by upregulation Myf5 transcription, causing myogenic precursors to stay in the quiescent state. This finding may explain the well-known anti-fatigue effect of salidroside, at least in part, due to the rapid recovery of exercise-induced damage in muscles by the increased reserve of the satellite cell pool.

Several signaling pathways have been reported to meditate the effect of salidroside. Salidroside protected *db/db* mice from insulin resistance by activating the AMPK/PI3K/AKT/GAS3β pathway in hepatocytes (Zheng et al., 2015). In bone marrow-derived endothelial progenitor cells, salidroside ameliorated oxidative stress-induced apoptosis by stimulating the AKT/mTOR/p70S6K and MAPK pathways (Tang et al., 2014). Additionally, salidroside has been shown to stimulate glucose uptake in skeletal muscle via AMPK activation (Li H.B. et al., 2008). Here, in C2C12 cells, we demonstrated that the inhibitory effect of salidroside on myogenesis is associated with the activation of Smad3. As one the most well-known pathways regulating muscle development, the TGF-β/Smad3 pathway inhibits muscle cell proliferation and differentiation both *in vivo* and *in vitro* (Liu et al., 2001; Langley et al., 2002; Zhu et al., 2004; Li X. et al., 2008; McFarlane et al., 2011). Liu et al. (2001) first reported that the TGF-β1 effector Smad3, but not Smad2, mediates the inhibition of myogenic differentiation mainly by repressing the activity of the transcriptional factor MyoD. Smad3 directly interacts with the bHLH domain of MyoD to antagonize the activity of MyoD, thereby inhibiting myogenic differentiation (Liu et al., 2001). Thus, the most likely mechanism by which salidroside promotes the transcription of Myf5 and inhibits myogenic differentiation is by upregulating the expression of p-Smad3. To confirm this possibility, we demonstrated that the exposure of the cells to SIS3, a SIS3 was able to block the activation of Myf5 by salidroside. This was confirmed using ChIP and luciferase reporter assays, which showed that Smad3 binding to the Myf5 promoter was essential for the enhancement of Myf5 expression induced by p-Smad3. Contrary to our results in muscle cells, salidroside has been reported to ameliorate pulmonary fibrosis by inhibiting the TGF-β1/Smad3 pathway (Tang et al., 2016). The conflicting effect may be due to the different tissue cells utilized in these studies. Further studies are needed to confirm whether salidroside has beneficial effects in treatment of muscular diseases.

In summary, our study for the first time demonstrated that salidroside inhibits the *in vitro* myogenesis of C2C12 cells by enhancing p-Smad3-induced Myf5 transcription and thus suggests a novel, intracellular, molecular mechanism linking extrinsic compound to the key myogenic transcriptional network. Although further investigation is needed to confirm whether the salidroside has the same effect *in vivo* as our *in vitro* findings, our study add new and important data for the assessment of the systemic effects of salidroside and may guide the future exploration of salidroside-based therapeutics for clinical applications.

## Author Contributions

XC conceived the project and designed the study. PZ, WL, HL, LW, JG, and FW carried out the experiments. PZ, WL, and HL analyzed the data. PZ and XC wrote the manuscript. All authors approved the final version of this manuscript.

## Conflict of Interest Statement

The authors declare that the research was conducted in the absence of any commercial or financial relationships that could be construed as a potential conflict of interest. The reviewer HL and handling Editor declared their shared affiliation.

## Footnotes

1 http://mulan.dcode.org/

## Supplementary Material

FIGURE S1 Salidroside has no cytotoxicity on C2C12 cells. C2C12 cells were treated with or without salidroside (25, 50, 100 μg/ml) and then cell viability was detected by MTT assay as described in Section “Materials and Methods” (*n* = 6). All data are shown as means ± SEM.

FIGURE S2 DN-Smad3 reversed the inhibitory effect of salidroside on C2C12 differentiation. C2C12 cells were either transfected with control (pCMV-myc) or DN-Smad3 plasmid and then treated with or without 50 μg/ml salidroside in DM for 120 h. Total RNA was then extracted and the levels of Myf5, MyoD and myogenin mRNA were quantitated by Real-time PCR analysis (*n* = 6). All data are shown as means ± SEM. ^∗∗^*p* < 0.01, ^##^*p* < 0.01 compared with groups as indicated by one-way ANOVA.

## Abbreviations

bHLH: basic helix-loop-helix
BMP: bone morphogenetic protein
ChIP: Chromatin Immunoprecipitation
DAPI: 4^′^,6^′^-diamidino-2-phenylindole
DM: differentiation medium
DMD: Duchenne muscular dystrophy
E-MHC: embryonic myosin heavy chain
GM: growth medium
HSCs: hematopoietic stem cells
IFA: immunofluorescence assay
MEF2: myocyte enhancer factor 2
MRFs: myogenic regulatory factors
PARP-1: poly (ADP-ribose) polymerase-1
p-Smad3: phosphorylated Smad3
RNAi: RNA interference
Sal: salidroside
siRNA: short interfering RNA
SIS3: specific inhibitor of p-Smad3
TGF-β: transforming growth factor beta
