# Supplementary figures and images for: Salidroside Inhibits Myogenesis by Modulating p-Smad3-Induced Myf5 Transcription
Source: Front Pharmacol. 2018 Mar 12;9:209. doi: 10.3389/fphar.2018.00209 (PMC5858519; doi:10.3389/fphar.2018.00209)

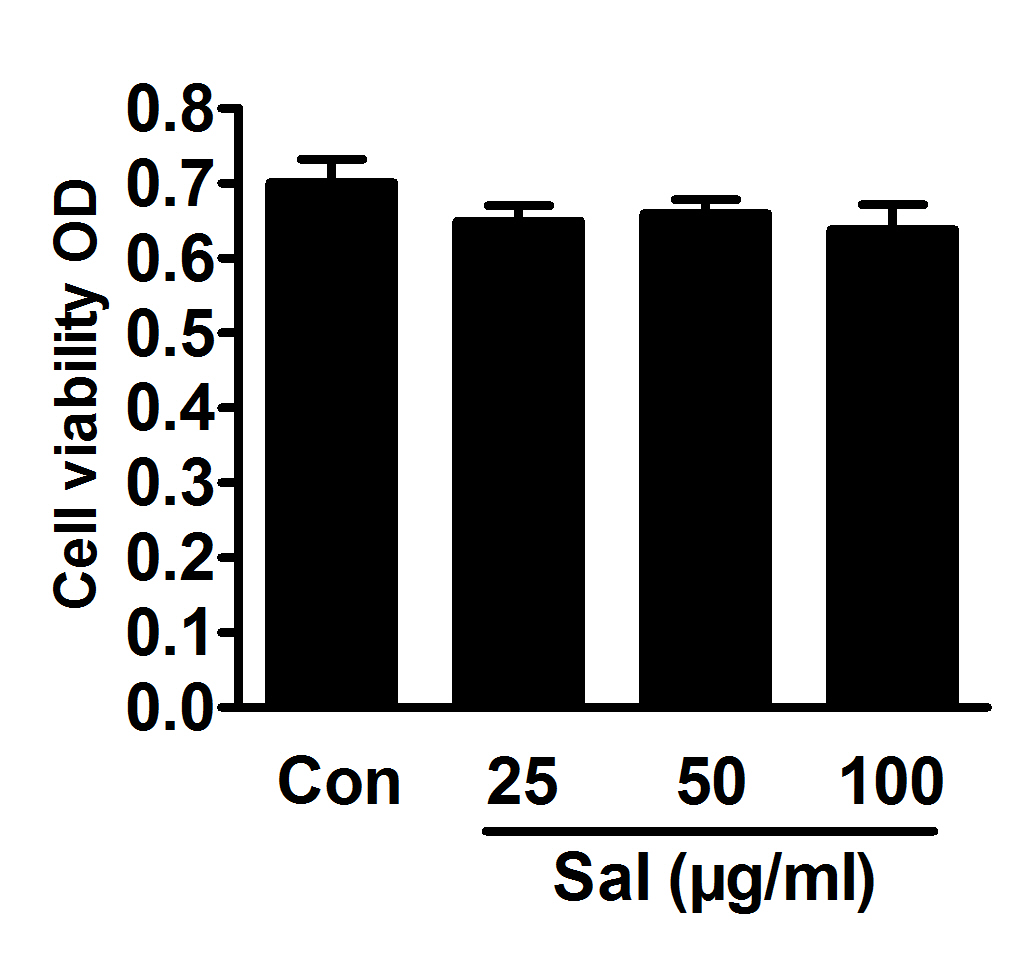

Supplement: FIGURE S1 — Salidroside has no cytotoxicity on C2C12 cells. C2C12 cells were treated with or without salidroside (25, 50, 100 μg/ml) and then cell viability was detected by MTT assay as described in Section “Materials and Methods” (n = 6). All data are shown as means ± SEM. [file Image_1.JPEG]

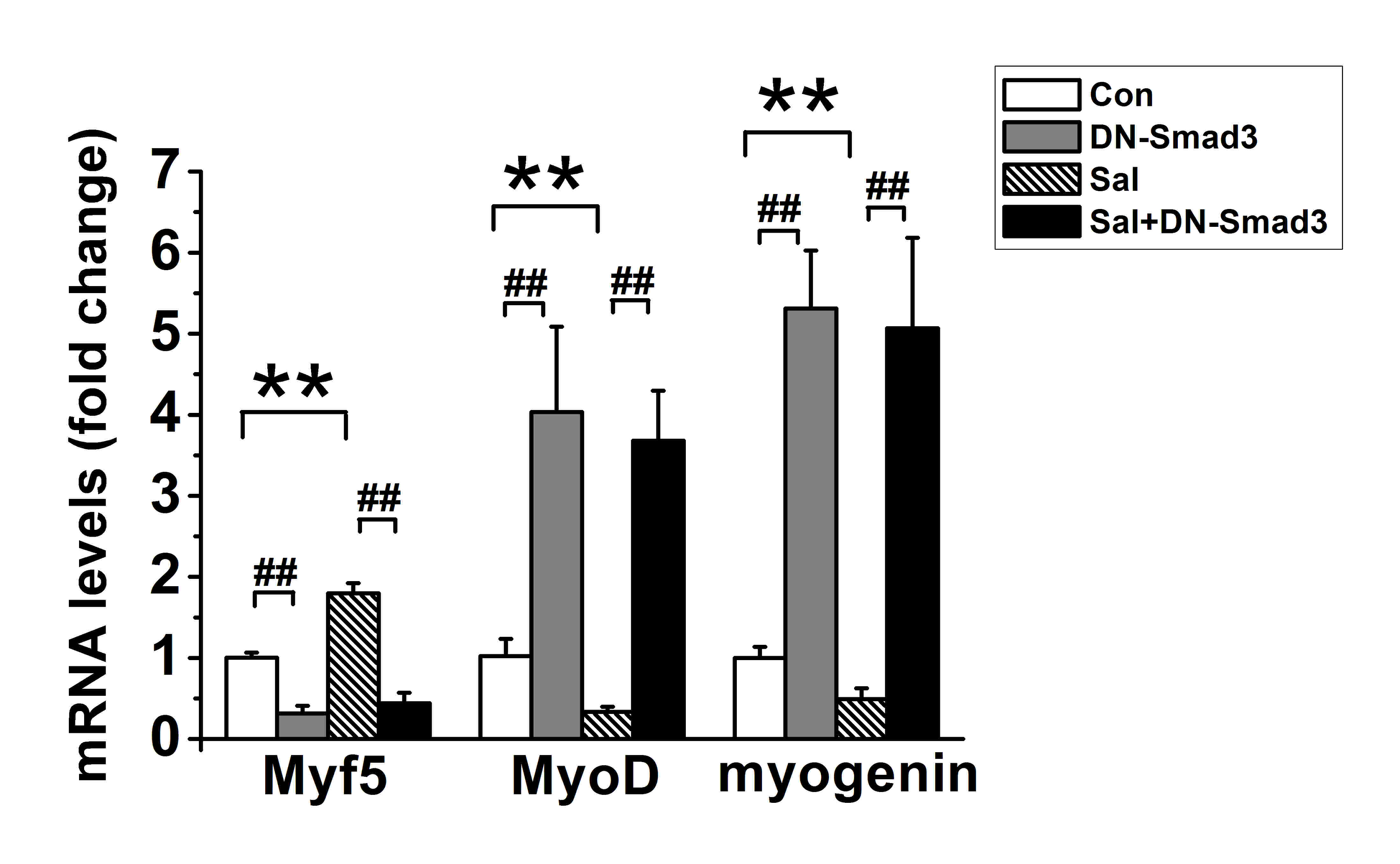

Supplement: FIGURE S2 — DN-Smad3 reversed the inhibitory effect of salidroside on C2C12 differentiation. C2C12 cells were either transfected with control (pCMV-myc) or DN-Smad3 plasmid and then treated with or without 50 μg/ml salidroside in DM for 120 h. Total RNA was then extracted and the levels of Myf5, MyoD and myogenin mRNA were quantitated by Real-time PCR analysis (n = 6). All data are shown as means ± SEM. ∗∗p < 0.01, ##p < 0.01 compared with groups as indicated by one-way ANOVA. [file Image_2.JPEG]
